# Continuous bronchoscopy during exercise in a pediatric patient: A case report

**DOI:** 10.14814/phy2.70543

**Published:** 2025-09-10

**Authors:** Andreas Kreutzer, Rebecca Brovina, Daniel Lim, John Marion Robertson

**Affiliations:** ^1^ Cook Children's Health Care System – Exercise Respiratory Center Prosper Texas USA; ^2^ University of North Texas Health Science Center – Texas College of Osteopathic Medicine Fort Worth Texas USA

**Keywords:** continuous laryngoscopy during exercise, double aortic arch, dyspnea, exercise‐induced laryngeal obstruction, tracheomalacia, vascular ring

## Abstract

Exercise‐induced respiratory symptoms limit physical activity and sport performance in adolescents. Etiologies include exercise‐induced bronchoconstriction, laryngeal obstruction, dysfunctional breathing, and in rarer cases, large airway obstruction and cardiac pathologies. Accurate diagnosis requires assessment during exercise that elicits the symptoms patients experience in the field. This is particularly important considering that misdiagnosis with asthma is common among those with laryngeal obstruction and leads to ineffective treatment and increased financial burden for patients and families. Continuous laryngoscopy is the gold standard for the evaluation of intermittent upper airway obstruction during exercise. Researchers recently established the feasibility of continuous bronchoscopy during exercise to assess the large airway in adults. We present the case of a 13‐year‐old female patient reporting dyspnea, chest tightness, and wheezing during exercise. A double aortic arch causing mild fixed tracheal compression did not appear to explain her symptoms. Vascular rings can cause tracheomalacia, another possible source of intermittent obstruction during exercise. We performed continuous bronchoscopy during exercise following continuous laryngoscopy during a cardiopulmonary exercise test. We found exercise‐induced laryngeal obstruction and ruled out tracheomalacia and other potential causes. To our knowledge, this was the first continuous bronchoscopy during exercise performed in a pediatric patient worldwide.

## 1 INTRODUCTION

Exercise‐induced respiratory symptoms, including dyspnea, wheezing, and stridor, are barriers to physical activity and sport performance in adolescents (Dantas et al., 2014; Glazebrook et al., 2006; Tilles, 2010). Dyspnea on exertion (DOE) is disproportionate breathlessness triggered by exercise or physical activity (Mukerji, 1990). Prevalence estimates of DOE and its causes come mostly from small studies using heterogeneous methodologies; thus, estimates presented in this report carry a degree of uncertainty. In studies by Johansson et al. and Ersson et al., 14% of a general adolescent cohort and 20% of athletes reported disproportionate shortness of breath during or after strenuous activity in the prior year (Ersson et al., 2020; Johansson et al., 2014).

Exercise‐induced bronchoconstriction (EIB) is the most common cause of DOE in adolescents. Prevalence estimates vary widely, in part due to different diagnostic testing modalities (Jackson et al., 2020). They range from 5% to 20% in the general population and 30% to 70% in elite athletes (Weiler et al., 2007). An under‐recognized cause of DOE, frequently misdiagnosed as EIB, is exercise‐induced laryngeal obstruction (EILO) (Bhatia et al., 2019; Hira, 2002; McFadden & Zawadski, 1996). EILO is an exercise‐triggered narrowing of the upper airway at the vocal cords (glottic), above the vocal cords (supraglottic), or both (mixed) (Liyanagedera et al., 2017). It occurs during inspiration and can be accompanied by stridor (Røksund et al., 2015). Some studies estimate that EILO is present in 5% to 8% the general population and in over 20% of athletes (Christensen et al., 2011; Clemm et al., 2022; Irewall et al., 2021; Jeppesen et al., 2024; Johansson et al., 2015; Nielsen et al., 2013). Evidence suggests that it develops at high exercise intensity (≥90% peak work capacity), minute ventilation (≥82% peak V̇E), and inspiratory flow rates (≥77% peak flow) in most patients (Brovina et al., 2025; Olin et al., 2016). However, there appears to be considerable variability between patients and EILO subtypes. Obstruction resolves quickly during recovery (often <60 s), requiring continuous laryngoscopy during exercise (CLE) for accurate diagnosis (Olin et al., 2016). Glottic and supraglottic obstruction is graded on a 0–3 point scale, with a score ≥2 indicating pathology (Maat et al., 2009). EILO is often comorbid with exercise‐induced dysfunctional breathing, an alteration in breathing patterns that results in symptoms (de Vos et al., 2019; Halvorsen et al., 2017). No gold standard diagnostic criteria for dysfunctional breathing exist, but aberrant patterns in the increase in tidal volume and respiratory rate in response to incremental exercise might indicate its presence (Ionescu et al., 2020).

In rare cases, exercise‐induced respiratory symptoms are caused by tracheal obstruction. This can result from tracheomalacia, excessive collapsibility of the tracheal wall due to disproportionate laxity of the posterior membrane or weak anterior cartilage (Wallis et al., 2019). Tracheomalacia can be secondary to a vascular ring (Wallis et al., 2019; Worhunsky et al., 2021). A reduction of cross‐sectional area >50% indicates pathology (Wallis et al., 2019). Symptoms of tracheal obstruction, including cough, wheezing, and dyspnea, can be triggered or worsened by exercise (Carden et al., 2005; Choo et al., 2013). The gold standard for evaluating tracheomalacia is dynamic flexible bronchoscopy under sedation using forced expiratory maneuvers (Corcoran et al., 2024; Majid et al., 2014). However, when assessing symptoms specific to exercise, the body position and higher expiratory pressures associated with this method compared to exercise can cause false positives (Williams et al., 2024). To address this, Williams et al. recently established the feasibility of a continuous bronchoscopy during exercise (CBE) in adults (Williams et al., 2024). In the present case report, we describe the first CBE in a pediatric patient. We prepared the report according to the CARE checklist; a completed copy is available as a supplementary file.

## 2 CASE PRESENTATION

A 13‐year‐old female presented to our institution with complaints of dyspnea, chest pain, chest tightness, and wheezing during exercise for the past 13 months. Her symptoms occurred primarily during soccer and gradually worsened over time, contributing to substantial weight gain. At presentation, her body mass index was 30.0 kg·m^−1^ (97th percentile). Table 1 shows patient characteristics and history.

**TABLE 1:** Patient demographics and medical history.

Demographics	
Age	13
Sex	Female
Height (cm)	168.5
Body mass (kg)	85.15
Sports	Soccer, volleyball, and karate
Medical history	
Hyperlipidemia	June 2020
Dyspnea, chest tightness, and wheezing on exertion	September 2023
Right‐sided double aortic arch with hypoplastic left arch	February 2024
Polycystic ovary syndrome	March 2024

**FIGURE 1:**
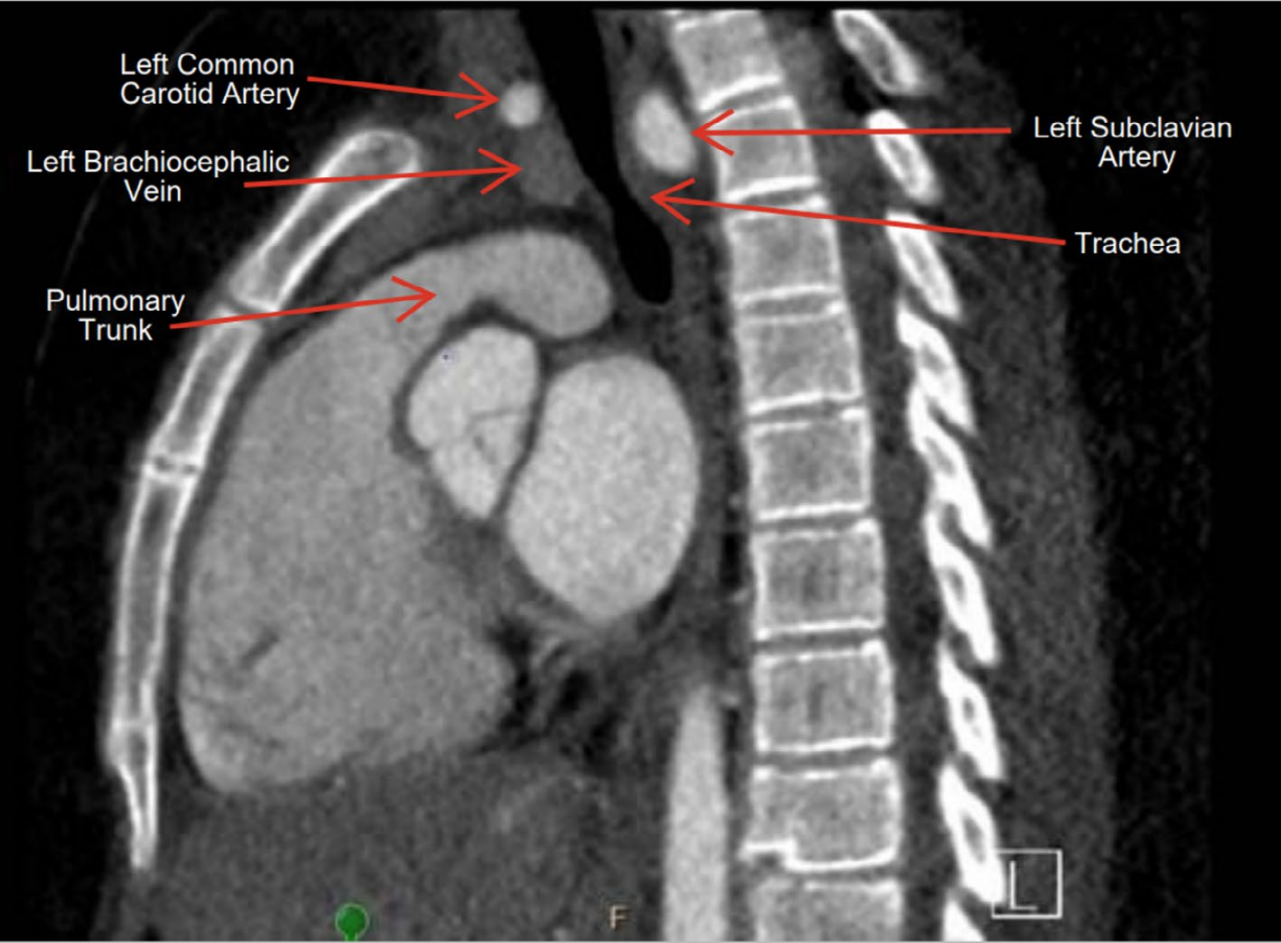
Computed tomography image showing fixed tracheal compression caused by the patient's double aortic arch.

She first reported DOE in September 2023. After detection of a heart murmur, an echocardiogram suggested a vascular ring. A computed tomography angiography confirmed a double aortic arch with hypoplastic left arch. This caused only mild (~40%) tracheal compression (Figure 1), making it an unlikely cause of her symptoms.

The patient saw pulmonology in April of 2024. Baseline spirometry was unremarkable, but post‐bronchodilator spirometry showed significant improvement (+10% FEV1), suggesting asthma. However, the patient's symptoms were unaffected by aerosolized albuterol before exercise. Therefore, she was referred to the Exercise Respiratory Center in May 2024.

To our knowledge, our center is one of three institutions in the United States routinely performing CLE in pediatric patients. The limited availability of CLE potentially contributes to the delay in diagnosis and the increased economic burden reported in patients with EILO (Fujiki et al., 2024; Walsted et al., 2021). Based on her symptoms and the possibility of the patient's vascular ring causing tracheomalacia, we performed a CBE following a CLE during cardiopulmonary exercise testing (CLE‐CPET). To our knowledge, this was the first pediatric CBE ever performed.

## 3 MATERIALS AND METHODS

On August 14, 2024, we performed a comprehensive evaluation, including CLE‐CPET, pre‐ and post‐exercise spirometry, and CBE. Prior to the CLE‐CPET, we applied two sprays of 0.05% solution of oxymetazoline per nostril for decongestion, followed by two 1.5‐milliliter aliquots of 2% viscous lidocaine to the right nostril for topical anesthesia. Thereafter, we inserted a flexible video‐rhino‐laryngoscope (KARL STORZ GMBH & CO. KG, Tuttlingen, Germany) and advanced it until the larynx was in view. The patient completed an incremental treadmill test to volitional exhaustion using a TMX428CP treadmill (Trackmaster, Newton, KS) and Ultima CardiO_2_ CPET system (MGC Diagnostics, Saint Paul, MN). The patient rated perceived exertion and DOE on a modified Borg Scale. Following the CLE‐CPET, the patient performed spirometry and rested for 30 min.

Before the CBE, we applied three two‐milliliter aliquots of 2% lidocaine solution to the vocal cords and four two‐milliliter aliquots to the trachea under direct observation with a flexible video‐bronchoscope (KARL STORZ GMBH & CO. KG, Tuttlingen, Germany), starting in the subglottic region and ending at the carina. We then reintroduced and advanced the scope until the area of tracheal compression was in view. The CBE followed the same exercise protocol as the CLE‐CPET, but did not capture cardiorespiratory data. Following the CBE, the patient completed a 5‐point Likert scale asking whether different parts of the procedure caused discomfort (“strongly disagree” to “strongly agree”).

## 4 RESULTS

The patient exercised for 8:15 min and 7:35 min in the CLE‐CPET and CBE, respectively. She achieved a peak oxygen consumption of 35.4 mL·kg·min^−1^ (85% predicted (Bongers et al., 2014)). Perceptual measures were similar during the CLE‐CPET and CBE (Table 2). Pre‐ and post‐exercise spirometry were normal, indicating EIB was absent.

**TABLE 2:** Cardiorespiratory and perceptual data during the CLE and CBE.

TM speed (mph)	V̇O_2_ (mL·kg·min^−1^)	HR (beats·min^−1^)	V_T_ (mL·breath^−1^)	RR (breaths·min^−1^)	RER	RPE		DOE	
	CLE	CLE	CLE	CLE	CLE	CLE	CBE	CLE	CBE
3.0	17.3	112	1146	29	0.73	1	1	1	1
4.5	28.7	161	1771	32	0.83	2	2	2	3
5.0	33.4	173	1717	40	0.89	4	4	4	5
5.5	34.1	178	1806	42	0.96	7	7	7	9
6.0	35.4	186	1709	42	1.01	9	10	10	10

Abbreviations: CBE, continuous bronchoscopy during exercise; CLE, continuous laryngoscopy during exercise; DOE, dyspnea on exertion; HR, heart rate; RER, respiratory exchange ratio; RPE, rating of perceived exertion; RR, respiratory rate; TM, treadmill; V̇O_2_, oxygen consumption; V_T_, tidal volume.

**FIGURE 2:**
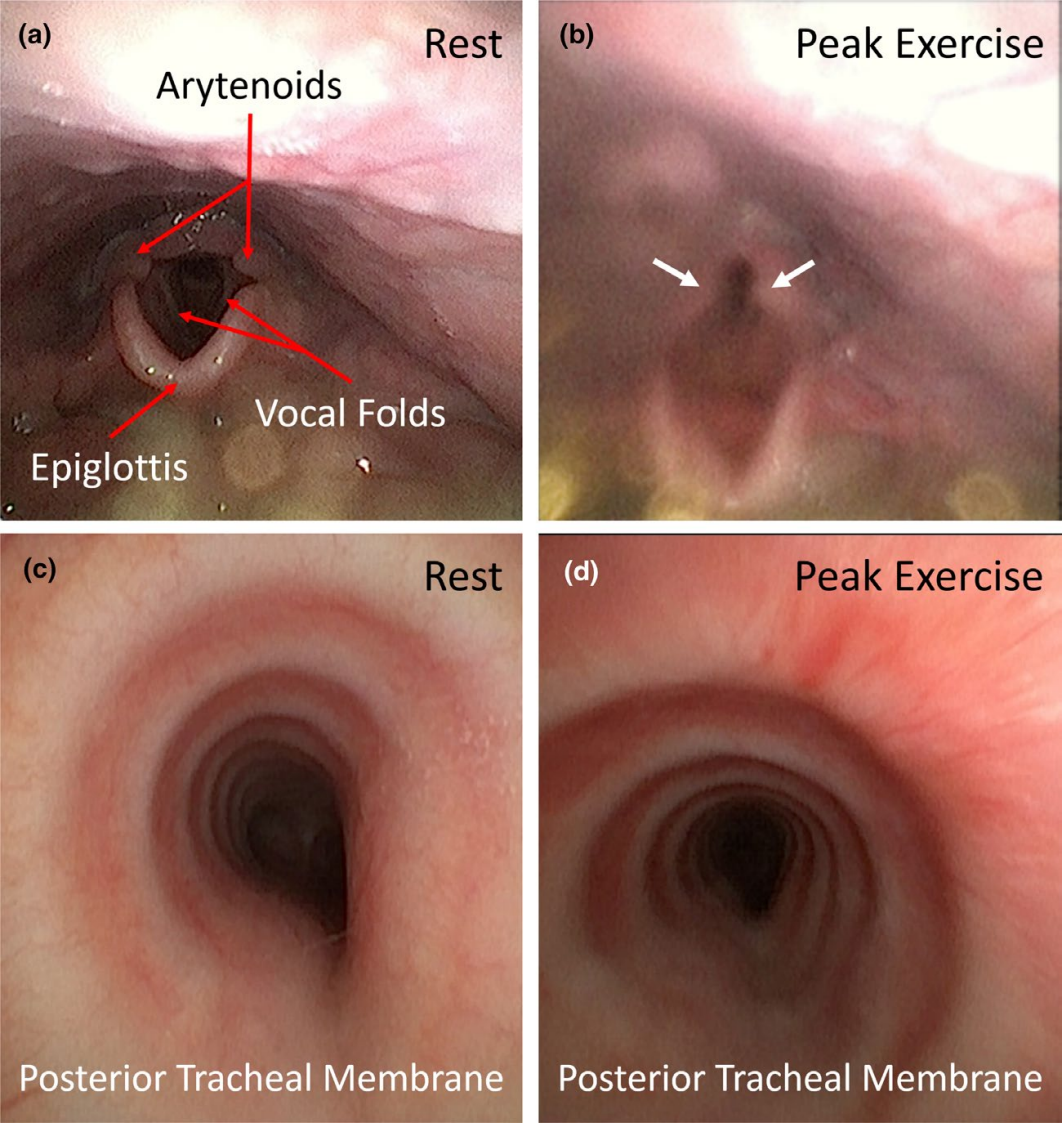
(a) Continuous laryngoscopy during exercise showing normal anatomy and function of the larynx during inspiration at rest. (b) Grade 2 supraglottic exercise‐induced laryngeal obstruction with arytenoids collapsing into the airway during inspiration at peak exercise intensity (white arrows). (c, d) Continuous bronchoscopy during exercise showing fixed tracheal compression at rest and exercise. No dynamic collapse during exhalation observed at peak intensity.

The CLE‐CPET revealed Grade 2 supraglottic EILO (Figure 2b). A normal pattern of tidal volume and respiratory rate and visual assessment during the CLE‐CPET suggested the absence of dysfunctional breathing. The CBE confirmed 40% compression of the distal trachea (Figure 2c) but did not show any expiratory collapse of the trachea (Figure 2d). A video of the test is available at https://www.youtube.com/shorts/H6oWzBURiVw.

### 4.1 Patient experience

On the 5‐point Likert scale, the patient reported discomfort from the use of lidocaine in the trachea (4), the placement of the bronchoscope (5), and exercise with the bronchoscope in place (4). She exhibited no discomfort due to the application of lidocaine in the nose (2), and a neutral rating (“neither agree nor disagree”) from lidocaine use on the vocal cords (3). A certified child life specialist attending the procedure noted that the patient was able to cope effectively and remain calm throughout all procedures.

### 4.2 Treatment

We referred the patient to a speech language pathologist to begin therapy for EILO focusing primarily on the Olin EILO biphasic inspiratory technique (Johnston et al., 2018). Treatment also includes diaphragmatic breathing retraining and release breathing techniques. As of May 2025, the patient is still undergoing therapy. At our center, EILO patients typically complete treatment within 4–6 weeks. However, this patient has struggled adhering to the schedule.

## 5 DISCUSSION

This case report demonstrates that a CLE‐CPET with pre‐ and post‐spirometry followed by CBE is feasible and tolerable in a pediatric patient. This is an important diagnostic modality because conditions like EILO, exercise‐induced dysfunctional breathing, and tracheomalacia are only detectable during exercise that replicates the symptoms patients experience in the field. In a study by Olin et al., supraglottic EILO resolved within 60 s following cessation of exercise in 84% of patients (Olin et al., 2016). In our patient, resolution occurred 72 s after the end of exercise, emphasizing the importance of continuous visualization of the larynx throughout exercise and recovery. While incompletely understood, EILO appears to have distinct underlying mechanisms that might differ between glottic and supraglottic cases, including anatomic and neurologic factors (Hilland et al., 2016; Hočevar‐Boltežar et al., 2017; Morrison et al., 1999; Reid & Hayatdavoodi, 2025; Walsted et al., 2018; Wysocki et al., 2008).

Supraglottic EILO might be triggered by high air flow rates creating inward and downward forces on the aryepiglottic folds causing inward collapse of the arytenoids (Halvorsen et al., 2017; Reid et al., 2023, 2024). These forces are up to 10‐fold greater at higher V̇E (180 L/min) when compared with lower V̇E (60 L/min) and appear to be moderated by the shape of an individual's hypopharynx (Reid & Hayatdavoodi, 2025). In the present patient, Grade 2 supraglottic obstruction developed at 55% of estimated maximal voluntary ventilation, 71% of peak V̇E, and 74% of peak inspiratory flow. While eucapnic voluntary hyperpnea (EVH) offers a way to elicit high V̇E at rest, investigations of its diagnostic capability for EILO are equivocal (Christensen & Rasmussen, 2013; Turmel et al., 2015). Christensen and Rasmussen stated that patients with Grade 2 or 3 obstruction during exercise experienced similar obstruction during EVH (Christensen & Rasmussen, 2013). However, their investigation of glottic angle and arytenoid rotation in a small subset of patients did not establish clear diagnostic capabilities of EVH. While they suggested that changes in glottic angle during EVH are very similar to those during exercise, their argument rests on a correlation analysis in six patients that appears to be heavily influenced by a single outlier. Conversely, Turmel et al. observed mild to severe supraglottic movement in 12 of 13 participants, but incomplete glottic adduction in only three (Turmel et al., 2015). Notably, they did not use the established grading system for EILO (Maat et al., 2009) and did not compare EVH findings to exercise. Thus, CLE remains the gold standard for diagnosing EILO.

Glottic EILO might be triggered by neurologic factors, including irritability of the larynx (Morrison et al., 1999). One hypothesis states that a hypersensitive glottic‐closure reflex contributes to paradoxical adduction of the vocal cords in response to high air flow during exercise (Halvorsen et al., 2017; Ikari & Sasaki, 1980; Morrison et al., 1999; Perkner et al., 1998). However, Hočevar‐Boltežar et al. found decreased laryngeal sensitivity in patients with EILO (Hočevar‐Boltežar et al., 2017). They suggested that the reflex threshold in EILO patients might be increased, but when it is reached, a strong closure ensues. To avoid any effect of the lidocaine applied to the vocal cords on laryngeal sensitivity during our evaluation of EILO, we administered topical laryngeal and tracheal anesthesia for the CBE after completion of the CLE‐CPET.

Tracheomalacia as a cause for exercise‐induced respiratory symptoms should be evaluated using CBE rather than dynamic bronchoscopy with forced breathing maneuvers. Expiratory pressures during these maneuvers can reach 170cmH_2_O, while typical peak values during CPET only reach 42cmH_2_O (Thomas et al., 1997; Tzelepis et al., 1997). This discrepancy could cause false positives when investigating exercise‐specific symptoms. In a study by Williams et al., 64% of healthy participants met criteria for excessive dynamic airway collapse (>50%) during dynamic magnetic resonance imaging and 24% during dynamic resting bronchoscopy; none of them met criteria during CBE, suggesting a high potential for false positives and misdiagnosis (Williams et al., 2024).

This case report shows that a comprehensive evaluation for EILO, EIB, exercise‐induced dysfunctional breathing, and tracheomalacia during a single outpatient procedural visit without sedation was feasible and tolerable in a pediatric patient.

## AUTHOR CONTRIBUTIONS

AK, JMR, and RB conceptualized, prepared, and performed the procedure. AK, RB, and DL drafted and revised the original manuscript. JMR critically reviewed and revised the manuscript and supervised the project. AK, RB, and JMR curated and analyzed the data. AK and RB created visualizations. All authors read and approved the final manuscript.

## FUNDING INFORMATION

None to declare.

## CONFLICT OF INTEREST STATEMENT

The authors have no conflicts of interest, financial or otherwise, to declare.

## ETHICS STATEMENT

Cook Children's does not require ethics review for single patient case reports. The patient's legally authorized representative provided consent for the publication of the patient's data and images.

## Data Availability

Data will be made available upon reasonable request.
